# Monomeric Nucleoprotein of Influenza A Virus

**DOI:** 10.1371/journal.ppat.1003275

**Published:** 2013-03-28

**Authors:** Sylvie Chenavas, Leandro F. Estrozi, Anny Slama-Schwok, Bernard Delmas, Carmelo Di Primo, Florence Baudin, Xinping Li, Thibaut Crépin, Rob W. H. Ruigrok

**Affiliations:** 1 UJF-EMBL-CNRS UMI 3265, Unit of Virus Host Cell Interactions, Grenoble, France; 2 CEA-CNRS-UJF UMR 5075, Institut de Biologie Structurale, Grenoble, France; 3 INRA UR 892, Virologie et Immunologie Moléculaires, Jouy en Josas, France; 4 Université de Bordeaux, Institut Européen de Chimie et de Biologie, ARNA laboratory, Pessac, France; 5 INSERM, U869, ARNA, Bordeaux, France; 6 European Molecular Biology Laboratory, Heidelberg, Germany; 7 MPI for Biology of Ageing, Bio-MS Facility, Cologne, Germany; Institut Pasteur, France

## Abstract

Isolated influenza A virus nucleoprotein exists in an equilibrium between monomers and trimers. Samples containing only monomers or only trimers can be stabilized by respectively low and high salt. The trimers bind RNA with high affinity but remain trimmers, whereas the monomers polymerise onto RNA forming nucleoprotein-RNA complexes. When wild type (wt) nucleoprotein is crystallized, it forms trimers, whether one starts with monomers or trimers. We therefore crystallized the obligate monomeric R416A mutant nucleoprotein and observed how the domain exchange loop that leads over to a neighbouring protomer in the trimer structure interacts with equivalent sites on the mutant monomer surface, avoiding polymerisation. The C-terminus of the monomer is bound to the side of the RNA binding surface, lowering its positive charge. Biophysical characterization of the mutant and wild type monomeric proteins gives the same results, suggesting that the exchange domain is folded in the same way for the wild type protein. In a search for how monomeric wt nucleoprotein may be stabilized in the infected cell we determined the phosphorylation sites on nucleoprotein isolated from virus particles. We found that serine 165 was phosphorylated and conserved in all influenza A and B viruses. The S165D mutant that mimics phosphorylation is monomeric and displays a lowered affinity for RNA compared with wt monomeric NP. This suggests that phosphorylation may regulate the polymerisation state and RNA binding of nucleoprotein in the infected cell. The monomer structure could be used for finding new anti influenza drugs because compounds that stabilize the monomer may slow down viral infection.

## Author Summary

The RNAs of negative strand RNA viruses are encapsidated by their specific viral nucleoproteins, forming helical nucleoprotein-RNA structures that are the template for transcription and replication. All these nucleoproteins have two activities in common: RNA binding and self-polymerisation, and it is likely that these activities are coupled. All these viruses have to keep their nucleoprotein from binding to cellular RNA and from polymerisation before viral RNA binding. The non-segmented viruses solve this by coding for a phosphoprotein that binds to the nucleoprotein, blocking both activities. The segmented viruses, such as influenza and Bunyaviruses, do not code for a phosphoprotein and need to solve this problem differently. Here we present the atomic structure of monomeric influenza virus nucleoprotein. Although the structures of the influenza virus and the Rift Valley Fever Virus (Bunya virus) nucleoproteins are different, there are functional similarities when the monomer and polymer structures are compared. Both nucleoproteins have a core structure that is identical in the monomer and the polymer. They contain a flexible arm that moves over to a neighbouring protomer in the polymer structure but that folds onto the core in the monomer structure, hiding the RNA binding groove in the Rift valley Fever Virus nucleoprotein and modifying the electrostatic potential of the RNA binding platform of the influenza virus protein.

## Introduction

Negative strand RNA viruses have an RNA genome in the opposite sense of that of messenger RNA. Therefore, the first viral activity after entering the host cell is transcription by the viral RNA-dependent RNA polymerase. The template for transcription is a complex between the viral RNA and the nucleoprotein (NP) that binds to the RNA sugar-phosphate backbone [1], [2]. NP is necessary for RNA elongation by the polymerase [3], [4]. However, its main function may be to separate the newly made mRNA from the template RNA because the infecting viral replication complexes do not contain helicases and purified influenza virus NP melts dsRNA [1].

Negative strand RNA viruses include non-segmented viruses like the *Rhabdoviridae* (ex. vesicular stomatitis virus (VSV) and rabies virus) and the *Paramyxoviridae* (ex. Sendai and measles virus) and segmented viruses like the *Arenaviridae* (Lassa fever virus), the *Bunyaviridae* (Rift Valley fever virus (RVFV)) and the *Orthomyxoviridae* (influenza viruses). When expressed in a transfected cell in the absence of other viral components, the nucleoproteins of most of these viruses bind to cellular RNA and form nucleoprotein-RNA complexes that are indistinguishable from the viral complexes [5]. The formation of such complexes results from two coupled activities of the nucleoproteins: RNA binding and self polymerisation. In infected cells, these nucleoproteins bind almost exclusively to their viral RNAs and, therefore, all these viruses have developed a mechanism to stop their NPs from binding to cellular RNA and from polymerizing. The non-segmented viruses code for another viral protein, the phosphoprotein (P), that binds with its N-terminal end to RNA-free nucleoprotein, indicated by N^0^ [6]–[8]. The structure of the N^0^P complex of VSV shows how the P binding site overlaps with the RNA binding groove on the nucleoprotein and with one of the sites involved in nucleoprotein polymerisation, thus blocking both activities [9].

The segmented viruses do not code for an equivalent of a phosphoprotein and solve the problem in different ways. The nucleoprotein of RVFV has been crystallised in two forms; as a monomer and as a hexameric ring [10], [11]. In the ring, two N-terminal α helices of NP swing out to the back of a neighbouring protomer for self polymerisation. Inside the ring there is a continuous positively charged surface that binds the RNA [12]. In the monomeric form the two N-terminal helices fold onto the positively charged surface of their own protomer. Thus, the monomeric, closed form avoids at the same time RNA binding and polymerisation. It is likely that, in the infected cell, a signal on the newly produced RNA or the polymerase itself changes the conformation of the nucleoprotein so that it binds to the viral RNA and polymerizes. The Lassa fever virus nucleoprotein was crystallised in its intact form that shows a C-terminal exonuclease domain [13], [14] and an N-terminal domain. The intact protein crystallised as a circular trimer with the C-terminal domain of one protomer binding to the N-terminal domain of the next one [13]. Neither of the domains had RNA bound. When the N-terminal domain was expressed alone it did bind RNA [15] and, compared with the closed structure of the intact protein, secondary structure elements had moved away to open the RNA binding cleft. The C-terminal domain may control the opening and closing of the RNA binding groove and a linear association of nucleoprotein protomers could permit the binding of RNA. Thus, although the structures of the RVFV and Lassa virus nucleoproteins are totally different, both proteins show inactive conformations with a closed RNA binding site and conformations in which secondary structure elements have moved away to open the RNA binding site [5]. For both proteins it was also suggested that polymerisation and RNA binding are coupled, like for the nucleoproteins of the non-segmented viruses.

The nucleoproteins of influenza A H1N1 and H5N1 crystallised as trimers [16], [17] and NP of influenza B virus as a tetramer [18]. In these structures, each protomer binds with a domain exchanged C-terminal tail loop (residues 402–428 for A/H1N1) in a groove on the core of a neighbouring protomer. On the opposite side of the loop, on the core of the protein, there is a large and shallow positively charged surface. Mutating residues on this surface lowers RNA binding [17]. NP purified from virus by CsCl gradient centrifugation exists in an equilibrium between monomers and oligomers going from trimers and tetramers to large structures resembling viral ribonucleoprotein complexes [19]. Although recombinant NP is generally considered to exist only as trimers [16], [17], it was recently shown that there exists an equilibrium between monomers and trimers/tetramers [20], [21]. This equilibrium is shifted to the oligomeric state at 300 mM salt whereas a stable population of monomers was found at 50 mM salt [20]. For some mutants like R416A and E339A the equilibrium is shifted to the monomeric form [16], [20], [22]–[24] whereas for the Y148A mutant the equilibrium is shifted to the trimeric form [20].

Here we show the structure of the monomeric R416A mutant and describe the RNA binding characteristics of monomeric wild type NP. In the infected cell NP may be kept monomeric by post translational modification. Mass spectroscopy analysis on NP isolated from viral RNP showed phosphorylation of serine 165. By mutagenesis we generated the S165D mutant to mimic this phosphorylation. S165D was monomeric with the same biophysical characteristics as the R416A mutant but showed high cooperativity for RNA binding at concentrations above the Kd.

## Results

**Figure 1:**
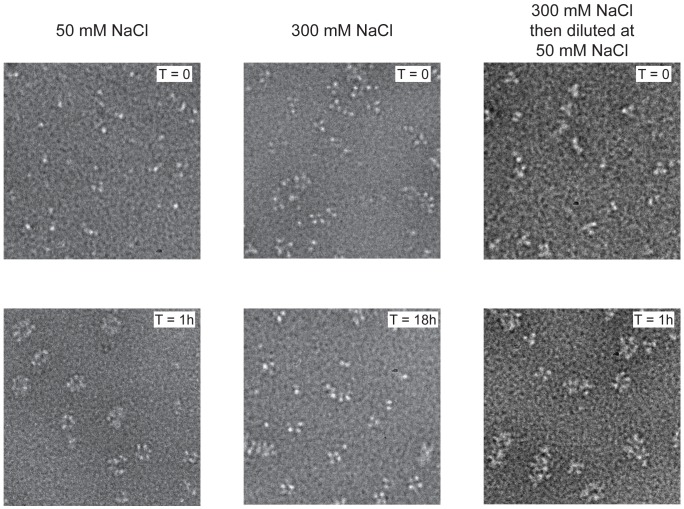
Kinetics of nucleoprotein-RNA ring formation. Negative stain electron micrographs of a 51 nucleotide RNA incubated with monomeric wt NP at 50 mM NaCl (left), oligomeric wt NP at 300 mM NaCl (middle) or oligomeric NP first at 300 mM NaCl which was subsequently diluted to 50 mM (right). The incubation times are indicated on the micrographs. T = 0 indicated the moment of mixing the RNA and NP and taking the first sample after 10–15 seconds. All magnifications are the same, the bar corresponds to 20 nm.

We previously showed that influenza virus NP can be stabilised as monomers in 50 mM salt and as trimers and tetramers in 300 mM salt [20] and wanted to further study their RNA binding behaviour. Wild type NP monomers and trimers/tetramers were incubated with an RNA oligonucleotide of 51 nucleotides [20]. Monomeric NP bound rapidly to the RNA and formed circular structures within 1 hour (Figure 1 left). These structures resemble circular recombinant mini RNPs [25]. The trimer/tetramer binds rapidly to RNA with a three-fold higher affinity than monomeric NP [20] but upon binding remained as trimers and tetramers up to 18 hours incubation (Figure 1 middle). However, when oligomeric NP was added to the RNA and then diluted to 50 mM NaCl, higher order polymers and rings formed within 1 hour like for the monomeric NP (Figure 1 right), most likely through dissociation of the oligomers upon which the monomers polymerised onto the RNA. In agreement with the results published in Tarus *et al.* [20] our results suggest that influenza NP is in equilibrium between monomers and oligomers but only monomers can form circular NP-RNA complexes.

**Figure 2:**
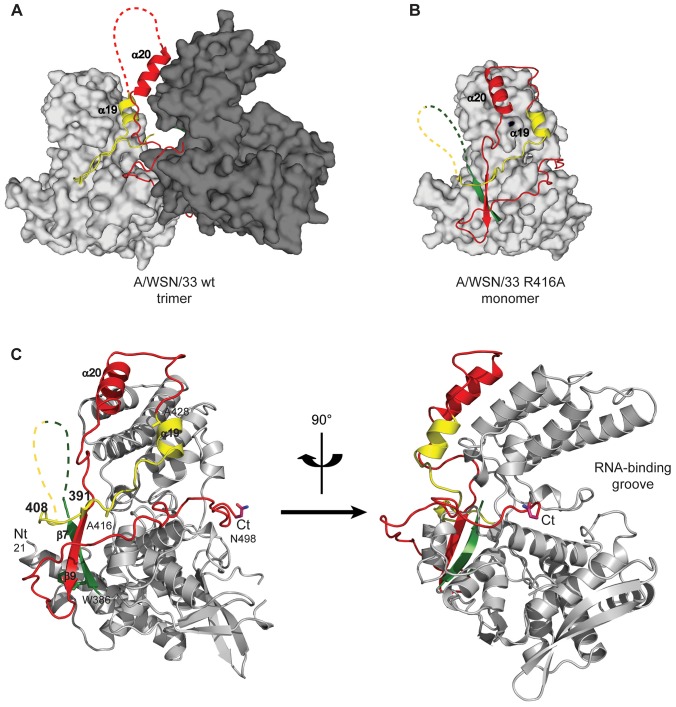
Structure of monomeric NP and comparison with the trimer structure. For the 4 panels residues 21–385 that have an identical structure between the monomer and the trimer are shown in grey, 386–401 in green, 402–428 in yellow and 429–498 in red. **A**. Two NP protomers in the trimer structure (PDB ID code 2IQH). The two bodies of the proteins (in atomic surface representation) are shown light and dark grey and for clarity only the exchange domain (in yellow) and C-terminal sequences (in red) of the dark grey protomer are shown. Residues 386–401(green) are behind the core of the dark grey protomer and not visible in this orientation. **B**. Monomer structure, the green, yellow and red parts are bound to their own protomer. **C**. Ribbon diagrams of two views of a single protomer in the monomer structure using the same colours as in A and B.

**Figure 3:**
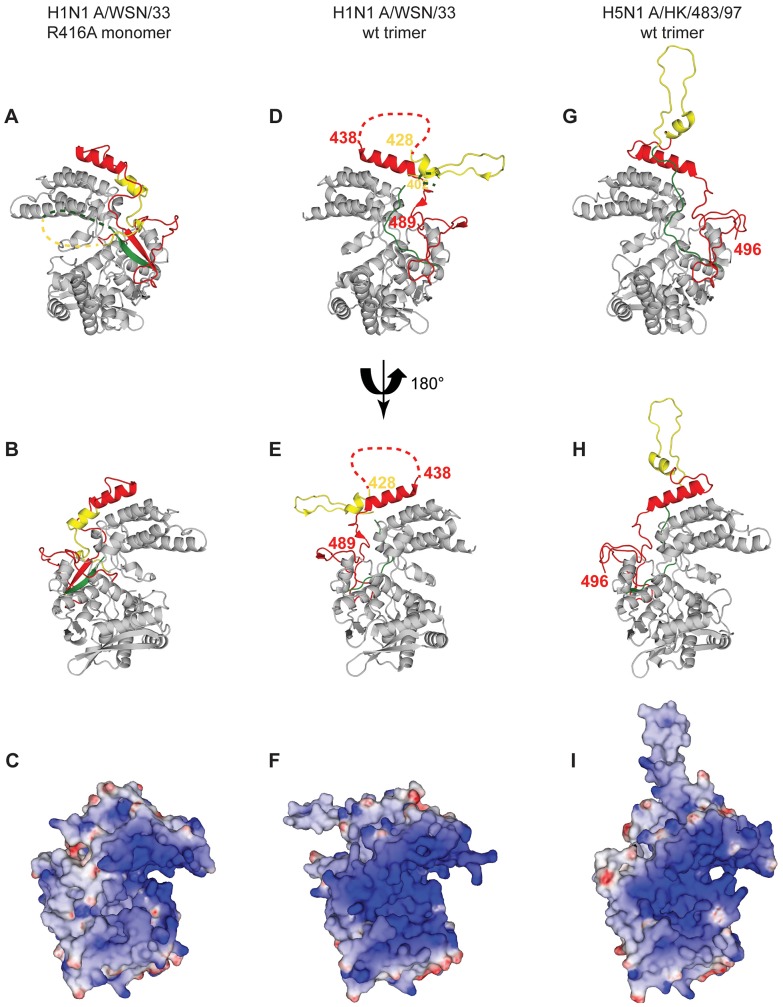
Comparison of the R416A monomer structure with the H1N1 and H5N1 wt trimer structures. **A and B.** Ribbon diagrams of two views (180° turn) of the monomer and **C** representation of the electrostatic surface of the monomer (from −10 kT/e (red) to +10 kT/e (blue)) calculated using DelPhi [52]. The views in **B** and **C** are the same. **D, E, F.** Similar representations for the wt H1N1 trimer structure (PDB ID code 2IQH). The beginning and end of the flexible loop between helices 19 and 20 are indicated as is the last visible residue at the C-terminus, residue 489. **G, H, I.** Similar representation for the H5N1 trimer structure (PDB ID code 2Q06) with last residue 496. Note that the exchange domains of the two trimeric structures point into different directions because these are bound to different neighbours in the trimer. Also note that the RNA binding surface of the monomer is less positively charged (less “blue”) than the trimeric structures due to the fact that the C-terminus is bound onto this surface whereas the C-terminal ends of the trimeric structures point away from this surface.

In order to study monomeric NP we tried to crystallise monomeric wt NP but we obtained the same crystals and structure as was obtained for the wt trimer [16]. Thus, the crystallisation conditions seem to push monomeric NP to form trimers in the crystal. We then crystallised the obligate monomeric R416A mutant of NP. Influenza A/WSN/33 R416A mutant NP was concentrated to 10 mg/ml and crystallised by vapour diffusion using the sitting drop method. Crystals with space group C222_1_ diffracted to 2.7 Å (Table 1). The structure was solved by molecular replacement starting from the structure of wt A/WSN/33 NP (PDB 2IQH) in which residues 400 to 498 had been removed. Even though the crystals of wt and mutant NP have the same space group, wt NP crystallises as a trimer whereas mutant NP crystallised as a monomer but with 3 molecules in the asymmetric unit; the cell dimensions are different and the positions of the monomers are different from those of the protomers in the trimer. The structure (Figures 2B, C and Figure S1 in Text S1) shows residues 21–391 and 408–498. Most of the mutant protein structure is identical to that of the wt, from residue 22 to 385, the rmsd is 1.09 Å for 364 aligned Cα's. From residue 386 onward the structure is different because the exchange domain folds into the groove of its own protomer rather than in the groove of a neighbouring protomer. Figure 2 shows the comparison of the structures of trimeric wt and monomeric R416A NP. The core of the protein is in grey because this part of the structure is the same for the wt trimer and the mutant monomer, residues 386 to 401 are in green, the trimer exchange domain (residues 402–428) is in yellow, and residues 429–498 are in red (Figure S1 in Text S1). Figure 2A shows the trimer structure and only the residues 402–489 of the dark grey protomer are shown. In the orientation shown residues 386–401, the green part, are behind its own protomer and, thus, not visible. In the wt trimer (Figures 2A, 3D, E and Figure S1 in Text S1), the “green” part of the chain forms a random coil to residue 402 of the exchange domain. Then, residues 402–421 (yellow, figure 2A) of the dark grey protomer form a hairpin that binds in a groove on the surface of the light grey protomer [16], [17]. This domain exchange is terminated by α-helix 19 that is also bound to the light grey protomer. Then the chain (in red) loops back to its own protomer starting with α-helix 20. The remainder of the C-terminal domain forms a random coil bound to the surface of the core of NP.

**Table 1:** Data collection and refinement statistics.

	NP R416A
**Data Collection**	
X-ray source	ESRF ID14-EH4
Wavelength (Å)	0.97625
Space group	*C*222_1_
Cell dimensions	
*a, b, c* (Å)	165.4, 285.4, 118.3
Resolution range(a) (Å)	40–2.7 (2.82–2.7)
Completeness(a) (%)	99.4 (99.7)
Rsym I(a) (b) (%)	9.9 (69.2)
I/σI	11.7 (1.9)
Total reflections(a)	254,298 (32,620)
Unique reflections(a)	77,415 (10,163)
Multiplicity(a)	3.3 (3.2)
**Refinement statistics**	
*R*-factor (%)	20.0
*R*_free_ (%)	24.9
Mean *B*-factor	
Protein	50.8
Ligand	50.4
No. Residue atoms	11 051
No. Ligand atoms	3
Ramachandran plot	
Allowed regions (%)	98.9
Disallowed regions (%)	1.1
R.m.s. deviations	
Bond lengths (Å)	0.015
Bond angles (°)	1.715

(a) Values in parentheses are for the highest-resolution shell.

(b) *R*sym (*I*) = [∑*_hkl_*∑*_i_*|<*I_hkl_*> - *I_hkl,i_*|]/[∑*_hkl_* ∑*_i_*|*I_hkl_*|], where *i* is the number of reflection *hkl*.

In the monomeric mutant protein (Figures 2B, C, 3A, B and Figure S1 in Text S1), residues 386–390 form a β-strand (β7) and residues 392–407 form a flexible loop that points towards the RNA binding surface. Then, from the mutated R416 up to α-helix 19 the chain binds in the groove on its own surface making very similar interactions as the domain exchange hairpin of the wt trimer. α-Helix 19 binds at exactly the same place as in the trimer structure but on its own protomer rather than on its neighbour. α-Helix 20 binds in exactly the same position to its own protomer in the monomer structure and in the trimer structure (rmsd of 0.5 Å between the two structures). The remaining “red” strand goes towards β-strand 7 to fold into β-strand 9 forming a two stranded β-sheet.

Figure 3 compares the H1N1 R416A monomer structure with the trimer structures of H1N1 and H5N1. The major difference between the H1N1 and H5N1 trimer structures is that the exchange domains don't bind to the same neighbours and, thus, point into different directions (Figure 3D and G). For trimeric H1N1 the last visible C-terminal residue is 489 and for H5N1 this is residue 496 and in both structures the C-termini point away from the RNA binding surface. However, in the structure of R416A, the chain is visible until the penultimate residue and points into the RNA binding surface, reducing the space for RNA binding and changing the electrostatic characteristics of this surface (compare figures 3C, F and I, blue is positively charged). At the other side of the monomer the 392–407 loop also points into the RNA binding surface. Although we could not model this loop because its density is missing, the RNA binding surface may be reduced from both sides.

Mutations in the domain exchange loop and in the surface groove in which the loop binds do not only influence the stability of the trimer but also the stability of the monomer. In the trimer structures, R416 makes an ionic bond with E339 and it was assumed that R416A and E339A formed monomers because this bond in the trimer was disrupted [16], [23], [26]. In the R416A structure, E339 makes hydrogen bonds with R461 and the mutated R416A points towards R461; if the wt monomer structure were the same as the R416A structure there could be a clash between arginines 416 and 461. Therefore, the R416A mutation may both stabilise the monomer and destabilise the trimer.

**Figure 4:**
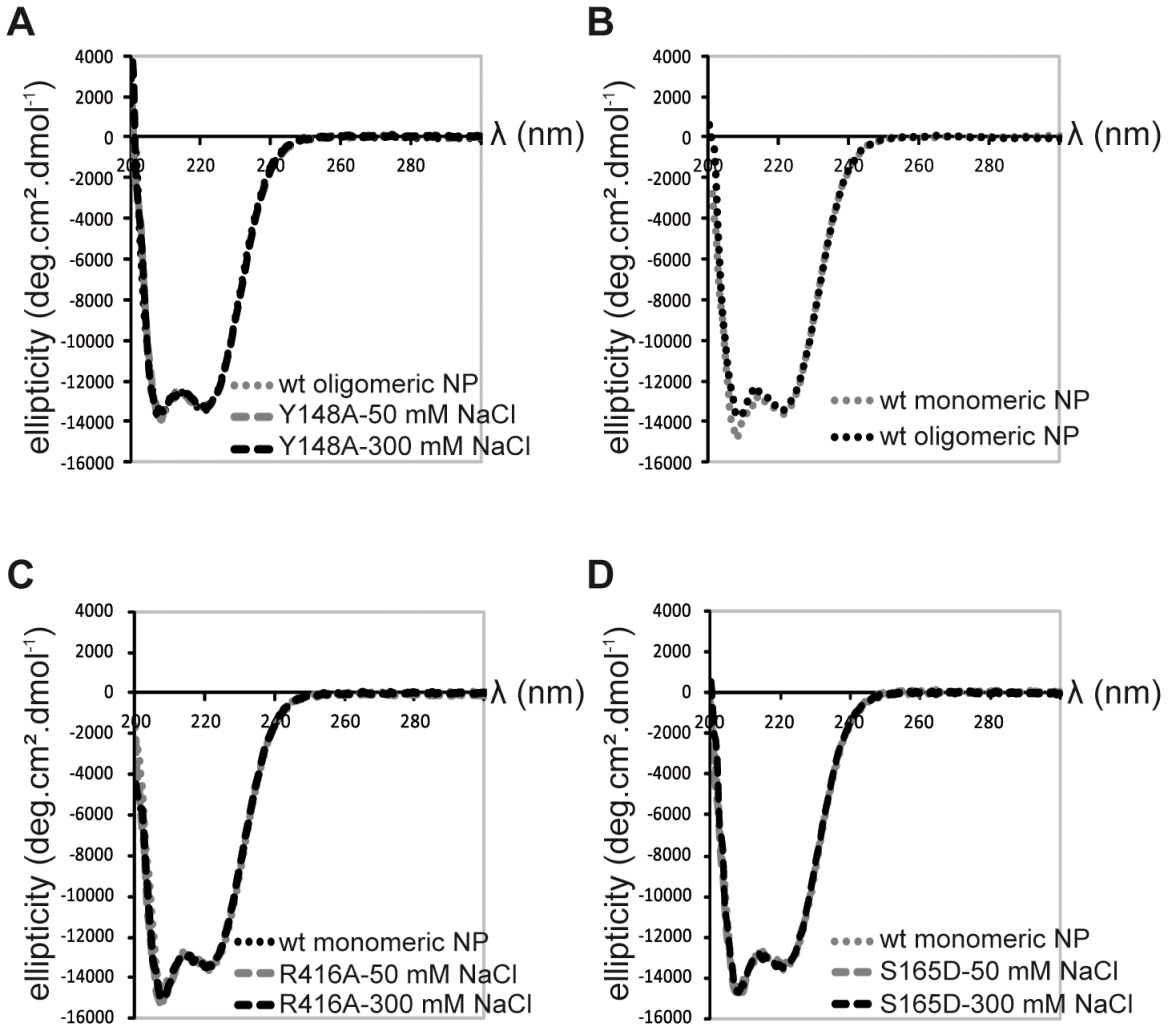
Circular dichroism on NP trimers and monomers. **A.** Trimeric wt and obligate trimeric Y148A mutant NP. **B.** Comparison of wt trimeric and monomeric NP. **C.** Monomeric wt and R416A NP. **D.** Monomeric wt and S165D NP.

Because we did not succeed in crystallising monomeric wt NP, we used a variety of biophysical methods to compare the wt and mutant monomers. Monomeric R416A and monomeric wt NP stabilised at low salt have an identical sedimentation behaviour with an S_20,w_ of 4.3 S [20]. This S-value corresponds to a hydrodynamic radius of 3.3 nm. This means that the exchange domain of the wild type monomer does not extend out in solution in the same conformation as in the oligomeric form but must be close to the core of the protein like in the R416A structure. The circular dichroism spectrum of the trimer form of the wt protein is identical to that of the obligate Y148A trimer, both at 50 and 300 mM NaCl (Figure 4A). The spectra could not be measured below 200 nm because of the high NaCl concentration and the secondary structure content could not reliably be determined. The spectra had a clear α-helical signature with a strong minimum at 222 nm. All spectra of monomeric NP (wt NP at 50 mM salt and R416A mutant NP at both 50 and 300 mM salt) were identical. They showed the same value at 222 nm as oligomeric NP but had a more pronounced minimum at 207 nm (Figure 4B and C). Each of these experiments was repeated with at least three independent protein preparations and always gave the same results. This indicates that all monomeric proteins have the same secondary structure content, slightly different from the oligomeric proteins. We also used CD to determine the melting temperature by heating the proteins while measuring the helical content at 208 and 222 nm. Again, the wt and mutant monomers showed an identical behaviour with an apparent denaturation midpoint value of 43.5±0.5°C (average of 6 independent measurements) whereas the wt trimer denatured at 47±0.5°C. All results for the monomeric wt and mutant proteins are identical and different from those for the oligomeric form thus, it is likely that monomeric wt NP has a similar structure as the R416A mutant NP.

**Figure 5:**
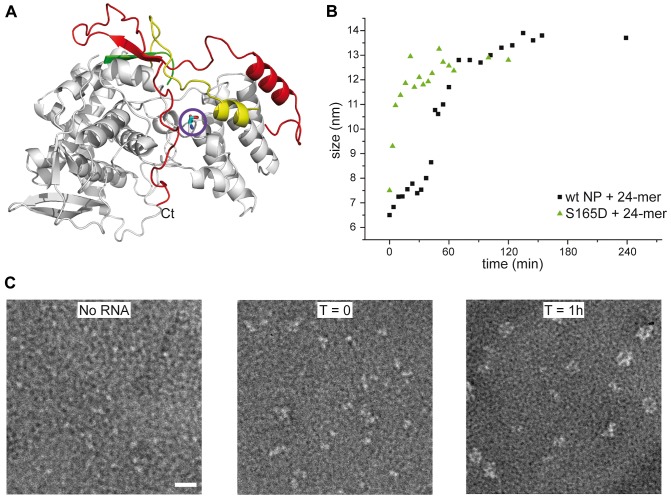
S165D mutant NP. **A.** Ribbon diagram in the same colors as in Figure 2 with serine 165 as a stick model encircled. **B.** Comparison of the kinetics of the formation of NP-RNA complexes using a 24 nt ribonucleotide by wt monomeric NP and the S165D mutant monitored by dynamic light scattering. **C.** Kinetics of formation of NP-RNA rings by the S165D mutant using a 51 nt ribonucleotide as followed by negative staining EM. In the T = 0 panel, with a sample preparation immediately after mixing the protein and the RNA, NP polymerisation onto the RNA can already be distinguished.

Because only monomeric NP forms RNP-like NP-RNA structures, NP newly produced in the infected cell should remain monomeric and free of nucleic acid until binding to viral RNA. However, accumulation of NP in the nucleus at physiological temperatures would likely result in the formation of trimers/tetramers and the affinity of monomeric NP for RNA is very high; Kd = 41 nM [20]. NP was reported to be phosphorylated on several serine residues [27], [28]. Phosphorylation was found to be a highly dynamic process and phosphorylated NP was also detected in RNPs [29]. We analyzed the NP isolated from influenza virus A/PR/8/34 RNPs by Mass Spectrometry. Several phosphorylated serines were detected, but only one residue is conserved in all A and B viruses: Serine 165. In the monomeric structure, this serine is situated at the interface between the two lobes of the core of NP, between α-helix 19 and residue Phe488, close to the C-terminus (Figure 5A). There is space for a phosphate group that could be stabilised by residue R267 nearby. In the trimer structure there does not seem to be enough space to locate a phosphate group because it would clash with main chain Ser407 and the negatively charged side chain of Glu405. We produced the S165D mutant mimicking the phosphorylation of Ser165. The mutant protein was monomeric at low and high salt when investigated by EM (Figure 5C), had the CD signature of a monomeric protein (Figure 4D), had a hydrodynamic radius of 3.3 nm and a thermal denaturation midpoint as determined by CD of 41±0.5°C and, thus, resembled the monomeric form of NP. The Kd for RNA of S165D was determined by surface plasmon resonance (SPR) using a 24 nt oligoribonucleotide [20] and by a filter binding assay using a radioactive panhandle RNA [1]. The SPR analysis gave a Kd of 730 nM (Figure S2 in Text S1); much higher than the Kd for wt monomeric NP of 41 nM [20]. The Kd derived from the filter binding experiment was in the same order of magnitude; 250 nM compared with 30 nM for wt recombinant NP and for NP isolated from virus (Figure S3 in Text S1). Thus, the affinity of the mutant was 10–20 times lower than that of wt protein. However, when studied by DLS at higher concentrations, the S165D mutant showed a much higher cooperativity upon RNA binding than wt NP. S165D NP polymerisation onto a 24-mer RNA oligonucleotide reached a plateau in 30 minutes compared to 2 hours for wt NP (Figure 5B). The same observation was made for the formation of NP-RNA rings as followed by negative staining EM. NP-RNA complexes formed immediately upon mixing of S165D NP with a 51 nt RNA oligo [20] (Figure 5C, T = 0) whereas complex formation with wt NP was slower (Figure 1). Thus, although the Kd for RNA is lower for the S165D mutant than for wt NP, the kinetics of assembly on RNA is more rapid.

## Discussion

Like for other negative strand RNA viruses, here we argue that influenza virus NP also exists in a monomeric form that is free of RNA and that only monomeric NP can form virus-like NP-RNA complexes. The structure of the monomer can perhaps best be described as self-inhibited in which the exchange domain that is involved in trimer formation takes up equivalent positions on its own core rather than on the core of a neighbouring protomer. A flexible loop formed by residues 392–407 and the C-terminus point towards the RNA binding surface which may be the reason for the lowered affinity for RNA, in particular for the obligate monomeric mutant R416A [20], [22], [24]. Wild type monomeric NP can bind oligonucleotides of 8 residues while remaining monomeric. However, when the RNA binding site is saturated with 24+ nucleotides [30], [31], the monomer oligomerises [20], possibly because the RNA pushes out secondary structure elements that stabilise the monomer. The stability of the wt monomer is enhanced by post-translational modification of serine 165. The biochemical behaviour of the monomeric S165D mutant is different with a lowered affinity for RNA but with an enhanced polymerisation at RNA concentrations above the Kd compared to wt monomeric NP. Recently, the lab of Ervin Fodor published the phosphoproteome of influenza A and B viruses [32]. Among various sites they also observed phosphorylation of S165. Recombinant virus with a S165A mutation could not be recovered indicating the importance of this phosphorylation site for the activity of the protein. Using RNP reconstitution experiments in 293 T cells they could measure transcription and replication for the S165A but not for a S165E mutant. As mentioned above the Ser to Asp mutation can be accommodated into the monomer structure but a mutation to Glu would lead to steric hindrance. While this paper was under review, two papers were published describing the structure of the intact viral RNP [33], [34]. Both papers describe that the distance between the NP protomers in the RNP is larger than the distance between the protomers in the trimers that are free from RNA [16] (Figure S4 in Text S1). This could imply that the insertion of the exchange domain into a neighbouring NP protomer in the RNP is not equivalent to that in the RNA-free trimer and this may be related to our findings that the S165D mutant does not form trimers but can polymerize onto RNA and that trimers can bind RNA but do not form NP-RNA rings.

Influenza NP binds to RNA without sequence specificity [1]. The only RNA sequence specificity for influenza virus proteins seems to reside in the polymerase that binds 5′ viral RNA and 5′-3′ panhandle structures [35]–[39]. In a recent model for influenza virus replication it was suggested that soluble, newly produced polymerase binds to the newly replicated 5′ end after which NP polymerises onto the elongated replicate [40], [41]. NP binds to the polymerase with a loop containing residues R204, W207 and R208 that is disordered in both the trimer and the monomer structures [42]. The mobility of this loop was recently suggested to influence RNA binding affinity [43]. It is possible that binding of NP to the polymerase leads to opening up of the RNA binding site after which the NP binds cooperatively to the primed NP-RNA site. Phosphorylation and de-phosphorylation of NP probably plays a regulatory role in vRNA encapsidation.

Because of its importance during the viral life cycle, NP is widely recognised as an antiviral target. Through high-throughput testing Kao *et al.* and Su *et al.* identified “nucleozin” that has antiviral activity and appears to aggregate NP in the cell leading to interference with nuclear import [44], [45]. A later crystal structure and mutational analysis showed that two NP trimers stick together into a hexamer by six nucleozin-derived molecules, with two complementary sites per NP protomer [46]. The crystal structure of the trimer has also been used for structure-aided drug design [26]. Peptides derived from the exchange domain (residues 402–428) interfere with polymerisation of NP and viral replication and molecules designed to interfere with the E339-R416 salt bridge have antiviral effect. The structure of the NP monomer presented here may serve for the design of new antiviral molecules. In particular, drugs that stabilise the monomer should have an antiviral effect.

## Materials and Methods

### Protein expression and purification of wt and mutant NP

The full-length NP gene of the H1N1 strain A/WSN/33 with a 6-His-tag at its C-terminal end was cloned in the pET22 vector (Novagen) under the control of a T7 promotor. The R416A, Y148A and S165D mutations were introduced by using *PfuUltra* DNA polymerase with the QuikChange II site-directed mutagenesis kit (Stratagene). *Escherichia coli* BL21 (DE3) cells carrying the plasmids were induced 4 hours by adding 1 mM isopropyl-β-D-thiogalactopyranoside (IPTG) at 37°C or 12 h at 28°C (mutant proteins) and collected by centrifugation. The pellet was resuspended and sonicated in a lysis buffer composed of 50 mM Tris at pH 7.4 with 300 mM NaCl, 15 to 30 mM imidazole, 1 M NDSB201 (Sigma), 5 mM β-ME and 5 mM MgCl_2_. The protein was purified by Ni^2+^ affinity chromatography (Ni-NTA, Qiagen) followed by a Heparin column (GE Healthcare). The protein was then dialyzed against 20 mM Tris pH 7.4 and 50 mM or 300 mM NaCl. The last purification step was size-exclusion chromatography using a Superdex S200 column. The protein was eluted in high or low salt according to the required polymerisation state. The protein concentration was determined by using the extinction coefficient ε = 55500 M^−1^.cm^−1^ at 280 nm.

### Crystallization, structure determination and refinement

The R416A mutant protein was crystallized by vapor diffusion using the sitting drop method. The crystals were obtained in 0.1 M Hepes pH 7.5, 1.2 M potassium sodium tartrate with a protein concentration of about 10 mg/ml. Data were collected at the ESRF (beamline ID14-4) and processed with the XDS package [47], [48]. The structure was solved by molecular replacement using the wild-type H1N1 nucleoprotein structure (PDB ID code 2IQH) as a model. Model building and refinement were performed using CCP4i suite program for crystallography (MOLREP, REFMAC, COOT) [49]. The coordinates have been deposited in the Protein Data Bank under PDB ID code 3zdp. The protein structure figures were made using PyMOL [50].

### Electron microscopy

Samples were applied between a carbon and a mica layer and negatively stained with 2% (w/v) sodium silicotungstate (pH 7.0). The carbon film was covered by a copper grid and air dried. Micrographs were recorded with a JEOL 1200 EX II microscope at 100 kV with a nominal magnification of 40,000×. Micrographs were taken on a 2K×2K CCD camera (Gatan Inc.). The RNA binding kinetics was performed with a protein solution at 100 µM and an RNA of 51 nucleotides [20] with a final ratio NP/RNA of 3/1. For the time points for EM analysis a fraction of the mix was diluted to have a final protein concentration between 10 and 20 µg/ml. The dilutions were made with a buffer with the same salt concentration except for the test of salt dilution on the monomer-oligomer equilibrium where the dilution was done with 20 mM Tris-HCl pH 7.4 in order to reduce the salt concentration from 300 to 50 mM NaCl.

### Dynamic light scattering

The measurements were performed on a Malvern nanosizer instrument thermostated at 20°C, in 20 mM Tris pH7.5, 50 or 300 mM NaCl according to the sample. The protein concentrations were in a range of 5 to 50 µM and for each sample, 12 to 18 scans were averaged. All experiments were repeated at least 3 times. The scattering intensity data were processed using the instrumental software to obtain the hydrodynamic radius and the size distribution of scatterers in each sample.

### Circular dichroism

Protein samples were centrifuged at 16000× g and the protein concentration in the supernatant was adjusted to 0.5 mg/ml. CD experiments were performed on a JASCO-810 spectrometer at 20°C with a 1 mm path-length quartz cell using a bandwidth of 1 nm, an integration time of 1 second and a scan rate of 50 nm/min. Each spectrum is the average of 10 scans. All spectra were corrected by subtracting the buffer spectrum acquired under the same conditions. All data were normalized to mean residue ellipticity. The melting temperature was obtained for each protein sample by measuring the CD signal at 208 nm and 222 nm from 20°C to 80°C every 2°C. At 80°C all proteins precipitated. The value of the denaturation midpoint (°C) is the average of at least 3 measurements on independent protein preparations.

### Determination of the dissociation equilibrium constant for binding of NP to RNA

#### Surface plasmon resonance experiments

The RNA binding kinetics were performed on a Biacore 3000 apparatus using streptavidin coated sensor chips (SA, Biacore) prepared as indicated by the manufacturer. Immobilization of the biotinylated 24 nt oligoribonucleotide [20] (100 to 200 RU) on the sensor chip was carried out in PBS buffer. The oligoribonucleotide was denatured at 80°C and renatured slowly at room temperature for one hour before each experiment. The S165D mutant was injected at concentrations between 0 and 8 µM. Measurements were conducted at 25°C and samples were injected at 25 µl/min flow rate. One flow cell left blank was used as a reference. To reduce the non-specific response to minimal values, 300 mM NaCl and 0.025% P20 surfactant were added to the running buffer. Because of non-specific polymerization of the S165D mutant NP, the sensorgrams could not be fitted by global analysis to determine the rate constants. The signals at the end of the injection were plotted as the function of the concentration. In order to determine an apparent dissociation equilibrium constant, a linear function that describes a non-specific binding mode was added to the standard Langmuir equation [51].

#### Filter binding experiments

The filter binding experiments were performed with a radiolabelled 81 nt panhandle RNA as described [1].

### Mass spectrometry identification of phosphorylation

NP from egg grown influenza A/PR/8/34 virus was cut out from a Coomassie-stained 1D SDS-PAGE gel and digested with Trypsin (Roche Applied Science). The digest was analyzed by LC-ESI-MS/MS with an Ion trap MS HCT ultra PTM Discovery System (Bruker Daltonics, Bremen, Germany) coupled with a Nano-LC 2D HPLC system (Eksigent, Dublin, CA, USA). A CapRod monolithic C18 column (100 µm×15 cm, Merck, Darmstadt, Germany) was used to separate the peptides. The gradient was 10–70% ACN within 20 min at 300 nl/min flow rate. The top two precursor ions were selected over m/z range from 400 to 1200 for fragmentation. Fragmentation was performed subsequently for 60 ms over 300 to 2000 m/z. The raw data were processed with DataAnalysis (Version 3.1, Bruker, Bremen Germany). The extracted MS/MS data were submitted to MASCOT (version 2.103, Matrix Science, London, UK) in-house server via Biotools (version3.0, Brucker, Bremen, Germany). Proteins were identified by searching the peptide lists against SwissProt. The following parameters were used: Taxonomy: Viruses; Enzyme: trypsin; Max Missed Cleavages: 2; Variable modifications: oxidation (M); carbamidomethyl (C); phospho (ST), phospho(Y); Peptide Mass Tolerance: ±0.5 Da: Fragment Mass Tolerance: ±0.5 Da.

## Supporting Information

Text S1 Supporting Figures S1, S2, S3, S4.
(PPTX)
